# The development and structural validity testing of the Person-centred Practice Inventory–Care (PCPI-C)

**DOI:** 10.1371/journal.pone.0303158

**Published:** 2024-05-10

**Authors:** Brendan George McCormack, Paul F. Slater, Fiona Gilmour, Denise Edgar, Stefan Gschwenter, Sonyia McFadden, Ciara Hughes, Val Wilson, Tanya McCance

**Affiliations:** 1 Faculty of Medicine and Health, Susan Wakil School of Nursing and Midwifery/Sydney Nursing School, The University of Sydney, Camperdown Campus, New South Wales, Australia; 2 Institute of Nursing and Health Research, Ulster University, Belfast, Northern Ireland; 3 Division of Nursing, Queen Margaret University, Edinburgh, Scotland; 4 Nursing and Midwifery Directorate, Illawarra Shoalhaven Local Health District, New South Wales, Australia; 5 Division of Nursing Science with Focus on Person-Centred Care Research, Karl Landsteiner University of Health Sciences, Krems, Austria; 6 Prince of Wales Hospital, South East Sydney Local Health District, New South Wales, Australia; University of Sharjah, UNITED ARAB EMIRATES

## Abstract

**Background:**

Person-centred healthcare focuses on placing the beliefs and values of service users at the centre of decision-making and creating the context for practitioners to do this effectively. Measuring the outcomes arising from person-centred practices is complex and challenging and often adopts multiple perspectives and approaches. Few measurement frameworks are grounded in an explicit person-centred theoretical framework.

**Aims:**

In the study reported in this paper, the aim was to develop a valid and reliable instrument to measure the experience of person-centred care by service users (patients)–The Person-centred Practice Inventory-Care (PCPI-C).

**Methods:**

Based on the ‘person-centred processes’ construct of an established Person-centred Practice Framework (PCPF), a service user instrument was developed to complement existing instruments informed by the same theoretical framework–the PCPF. An exploratory sequential mixed methods design was used to construct and test the instrument, working with international partners and service users in Scotland, Northern Ireland, Australia and Austria. A three-phase approach was adopted to the development and testing of the PCPI-C: *Phase 1 –Item Selection*: following an iterative process a list of 20 items were agreed upon by the research team for use in phase 2 of the project; *Phase 2 –Instrument Development and Refinement*: Development of the PCPI-C was undertaken through two stages. Stage 1 involved three sequential rounds of data collection using focus groups in Scotland, Australia and Northern Ireland; Stage 2 involved distributing the instrument to members of a global community of practice for person-centred practice for review and feedback, as well as refinement and translation through one: one interviews in Austria. *Phase 3*: *Testing Structural Validity of the PCPI-C*: A sample of 452 participants participated in this phase of the study. Service users participating in existing cancer research in the UK, Malta, Poland and Portugal, as well as care homes research in Austria completed the draft PCPI-C. Data were collected over a 14month period (January 2021-March 2022). Descriptive and measures of dispersion statistics were generated for all items to help inform subsequent analysis. Confirmatory factor analysis was conducted using maximum likelihood robust extraction testing of the 5-factor model of the PCPI-C.

**Results:**

The testing of the PCPI-C resulted in a final 18 item instrument. The results demonstrate that the PCPI-C is a psychometrically sound instrument, supporting a five-factor model that examines the service user’s perspective of what constitutes person-centred care.

**Conclusion and implications:**

This new instrument is generic in nature and so can be used to evaluate how person-centredness is perceived by service users in different healthcare contexts and at different levels of an organisation. Thus, it brings a service user perspective to an organisation-wide evaluation framework.

## Introduction

Person-centred healthcare focuses on placing the beliefs and values of service users at the centre of decision-making and creating the context for practitioners to do this effectively. Person-centred healthcare goes beyond other models of shared decision-making as it requires practitioners to work with service users (patients) as actively engaged partners in care [1]. It is widely agreed that person-centred practice has a positive influence on the care experiences of all people associated with healthcare, service users and staff alike. International evidence shows that person-centred practice has the capacity to have a positive effect on the health and social care experiences of service users and staff [1–4]. Person-centred practice is a complex health care process and exists in the presence of respectful relationships, attitudes and behaviours [5]. Fundamentally, person-centred healthcare can be seen as a move away from neo-liberal models towards the humanising of healthcare delivery, with a focus on the development of individualised approaches to care and interventions, rather than seeing people as ‘products’ that need to be moved through the system in an efficient and cost-effective way [6].

Person-centred healthcare is underpinned by philosophical and theoretical constructs that frame all aspects of healthcare delivery, from the macro-perspective of policy and organisational practices to the micro-perspective of person-to-person interaction and experience of healthcare (whether as professional or service user) and so is promoted as a core attribute of the healthcare workforce [1,7]. However, Dewing and McCormack [8] highlighted the problems of the diverse application of concepts, theories and models all under the label of person-centredness, leading to a perception of person-centred healthcare being poorly defined, non-specific and overly generalised. Whilst person-centredness has become a well-used term globally, it is often used interchangeably with other terms such as ’woman-centredness’ [9], ’child-centredness’ [10], ’family-centredness’ [11], ’client-centredness’ [12] and ’patient-centredness’ [13]. In their review of person-centred care, Harding et al [14] identified three fundamental ‘stances’ that encompass person-centred care—*Person-centred care as an overarching grouping of concepts*: includes care based on shared-decision making, care planning, integrated care, patient information and self-management support; *Person-centred care emphasising personhood*: people being immersed in their own context and a person as a discrete human being; *Person-centred care as partnership*: care imbued with mutuality, trust, collaboration for care, and a therapeutic relationship.

Harding et al. adopt the narrow focus of ’care’ in their review, and others contend that for person-centred care to be operationalised there is a need to understand it from an inclusive whole-systems perspective [15] and as a philosophy to be applied to all persons. This inclusive approach has enabled the principles of person-centredness to be integrated at different levels of healthcare organisations and thus enable its embeddedness in health systems [16–19]. This inclusive approach is significant as person-centred care is impossible to sustain if person-centred cultures do not exist in healthcare organisations [20,21].

McCance and McCormack [5] developed the Person-centred Practice Framework (PCPF) to highlight the factors that affect the delivery of person-centred practices. McCormack and McCance published the original person-centred nursing framework in 2006. The Framework has evolved over two decades of research and development activity into a transdisciplinary framework and has made a significant contribution to the landscape of person-centredness globally. Not only does it enable the articulation of the dynamic nature of person-centredness, recognising complexity at different levels in healthcare systems, but it offers a common language and a shared understanding of person-centred practice. The Person-centred Practice Framework is underpinned by the following definition of person-centredness:

*[A]n approach to practice established through the formation and fostering of healthful relationships between all care providers*, *service users and others significant to them in their lives*. *It is underpinned by values of respect for persons*, *individual right to self-determination*, *mutual respect and understanding*. *It is enabled by cultures of empowerment that foster continuous approaches to practice development* [16].

**Fig 1:**
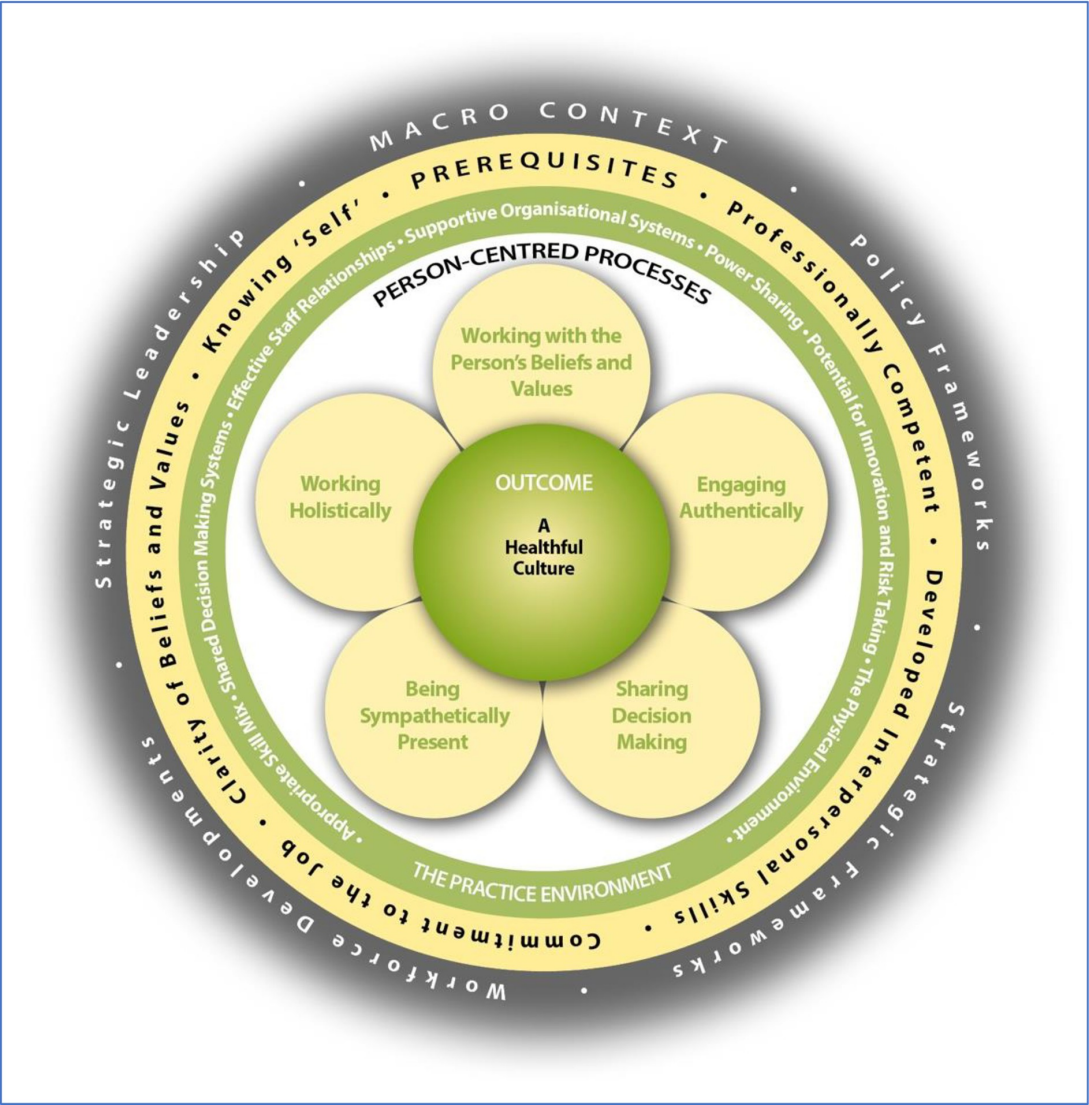
Person-centred practice framework.

The Person-centred Practice Framework (Fig 1) comprises five domains: the *macro context* reflects the factors that are strategic and political in nature that influence the development of person-centred cultures; *prerequisites* focus on the attributes of staff; the *practice environment* focuses on the context in which healthcare is experienced; the *person-centred processes* focus on ways of engaging that are necessary to create connections between persons; and the *outcome*, which is the result of effective person-centred practice. The relationships between the five domains of the Person-centred Practice Framework are represented pictorially, that being, to reach the centre of the framework, strategic and policy frames of reference need to be attended to, then the attributes of staff must be considered as a prerequisite to managing the practice environment and to engaging effectively through the person-centred processes. This ordering ultimately leads to the achievement of the outcome–the central component of the framework. It is also important to recognise that there are relationships and there is overlap between the constructs within each domain.

In 2015, Slater et al. [22] developed an instrument for staff to use to measure person centred practice- the Person-centred Practice Inventory- Staff (PCPI-S). The PCPI-S is a 59-item, self-report measure of health professionals’ perceptions of their person-centred practice. The items in the PCPI-S relate to seventeen constructs across three domains of the PCPF (prerequisites, practice environment and person-centred processes). The PCPI-S has been widely used, translated into multiple languages and has undergone extensive psychometric testing [23–28].

No instrument exists to measure service users’ perspectives of person-centred care that is based on an established person-centred theoretical framework or that is designed to compare with service providers perceptions of it. In an attempt to address this gap in the evidence base, this study set out to develop such a valid and reliable instrument. The PCPI-C focuses on the person-centred processes domain, with the intention of measuring service users’ experiences of person-centred care. The person-centred processes are the components of care that directly affect service users’ experiences. The person-centred processes enable person-centred care outcomes to be achieved and include working with the person’s beliefs and values, sharing decision-making, engaging authentically, being sympathetically present and working holistically. Based on the ‘person-centred processes’ construct of the PCPF and relevant items from the PCPI-S, a version for service users was developed.

This paper describes the processes used to develop and test the instrument–The Person-centred Practice Inventory-Care (PCPI-C). The PCPI-C has the potential to enable healthcare services to understand service users’ experience of care and how they align with those of healthcare providers.

## Materials and methods

The aim of this research was to develop and test the face validity of a service users’ version of the person-centred practice inventory–The Person-centred Practice Inventory-Care.

**Fig 2:**
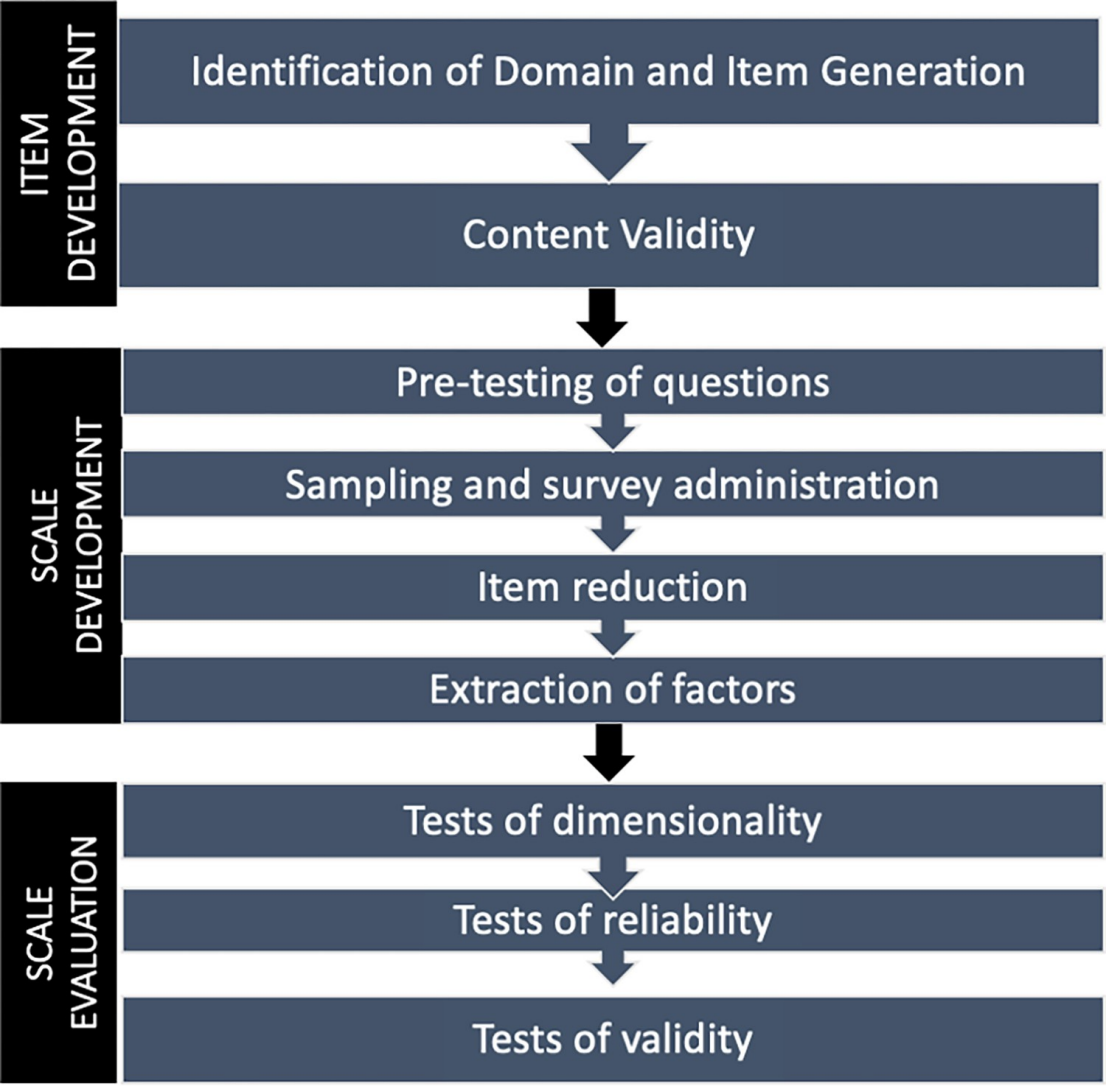
The phases in developing and testing a psychometrically valid instrument (adapted from Boateng et al 2018) [29].

The development and testing of the instrument was guided by the instrument development principles of Boateng et al [29] (Fig 2) and reported in line with the COSMIN guidelines for instrument testing [30,31]. An exploratory sequential mixed methods design was used to construct and test the instrument [29,30] working with international partners and service users. A three-phase approach was adopted to the development and testing of the PCPI-C. As phases 1 and 2 intentionally informed phase 3 (the testing phase), these two phases are included here in our description of methods.

### Ethical approval

Ethics approval was sought and gained for each phase of the study and across each of the participating sites. For phase 2 of the study, a generic research protocol was developed and adapted for use by the Scottish, Australian and Northern Irish teams to apply for local ethical approval. In Scotland, ethics approval was gained from Queen Margaret University Edinburgh Divisional Research Ethics Committee; in Australia, ethics approval was gained from The University of Wollongong and in Northern Ireland ethics approval was gained from the Research Governance Filter Committee, Nursing and Health Research, Ulster University. For phase 3 of the study, secondary analysis of an existing data set was undertaken. For the original study from which this data was derived (see phase 3 for details), ethical approval was granted by the UK Office of Research Ethics Committee Northern Ireland (ORECNI Ref: FCNUR-21-019) and Ulster University Research Ethics Committee. Additional local approvals were obtained for each partner site as required. In addition, a data sharing agreement was generated to facilitate sharing of study data between European Union (EU) sites and the United Kingdom (UK).

### Phase 1 –Item selection

An initial item pool for the PCPI-C was identified by <author initials to be added after peer-review> by selecting items from the ‘person-centred processes’ sub-scale of the PCPI-S (Table 1). Sixteen items were extracted, and the wording of the statements was adjusted to reflect a service-user perspective. Additional items were identified (n = 4) to fully represent the construct from a service-user perspective. A final list of 20 items was agreed upon and this 20-item questionnaire was used in Phase 2 of the instrument development.

**Table 1:** Person-centred processes domain: Construct definitions, PCPI-S items & PCPI-C items.

Construct Definition	Items chosen from the PCPI-S	PCPI-C Items
***Working with the person’s beliefs & values*** Having a clear picture of what the person’s values about his/her life and how he/she makes sense of what is happening from their individual perspective, psychosocial context and social role	• I integrate my knowledge of the person into care delivery. • I work with the person within the context of their family and carers. • I seek feedback on how people make sense of their care experience. • I encourage the people to discuss what is important to them.	• Staff make an effort to get to understand what is important to me • Staff use my personal experiences to build a relationship with me • I feel able to give staff feedback about my experience of being cared for • I feel able to say to staff what is important to me
***Sharing decision-making*** Engaging persons in decision-making by considering values, experiences, concerns and future aspirations	• I include the family in care decisions where appropriate and/or in line with the person’s wishes. • I work with the person to set health goals for their future. • I enable people receiving care to seek information about their care from other healthcare professionals	• Staff involve me in making decisions about my care • My family are included in decisions about my care when I want them to be. • Staff help me to set realistic goals • Staff help me to express my concerns about my treatment and care • I get all the information I need
***Engaging authentically*** The connectedness between people, determined by knowledge of the person, clarity of beliefs and values, knowledge of self and professional expertise.	• I try to understand the person’s perspective. • I seek to resolve issues when my goals for the person differ from theirs perspectives. • I engage people in care processes where appropriate.	• When we don’t agree about my care staff try to find common ground • Staff listen to me and hear what I have to say about my care • Staff don’t take for granted they know what is best for me • Staff connect with me as a person
***Being sympathetically present*** An engagement that recognises the uniqueness and value of the person, by appropriately responding to cues that maximise coping resources through the recognition of important agendas in their life	• I actively listen to people receiving care to identify unmet needs. • I gather additional information to help me support the people receiving care. • I ensure my full attention is focused on the person when I am with them.	• Staff respond compassionately when I am upset or unhappy • Staff give me their full attention when they are with me • Staff use what they know about me as a person in my care
***Working holistically*** *Ways of connecting that pay attention to the whole person through the integration of physiological*, *psychological*, *sociocultural*, *developmental and spiritual dimensions of persons*	• I strive to gain a sense of the whole person. • I assess the needs of the person, taking account of all aspects of their lives. • I deliver care that takes account of the whole person.	• I feel cared for • Staff take account of all aspects of my life • Staff consider my home environment in meeting my care needs • Staff understand my family circumstances when caring for me

### Phase 2 –Instrument development and refinement

Testing the validity of PCPI-C was undertaken through three sequential rounds of data collection using focus groups in Scotland, Australia and Northern Ireland. The purpose of these focus groups was to work with service users to share and compare understandings and views of their experiences of healthcare and to consider these experiences in the context of the initial set of PCPI-C items generated in phase 1 of the study. These countries were selected as the lead researchers had established relationships with healthcare partners who were willing to host the research. The inclusion of multiple countries provided different perspectives from service users who used different health services. In Scotland, a convenience sample of service users (n = 11) attending a palliative care day centre of a local hospice was selected. In Australia a cancer support group for people living with a cancer diagnosis (n = 9) was selected and in Northern Ireland, people with lived experience who were attending a community group hosted by a Cancer Charity (n = 9) were selected. All service users were current users of healthcare and so the challenge of memory recall was avoided. The type of conditions/health problems of participants was not the primary concern. Instead, we targeted persons who had recent experiences of the health system. The three centres selected were known to the researchers in those geographical areas and relationships were already established, which helped with gaining access to potential participants. Whilst the research team had potential access to other centres in each country, it was evident at focus group 3 that no significant new issues were being identified from the participants and thus we agreed to not do further rounds of refinement.

**Fig 3:**
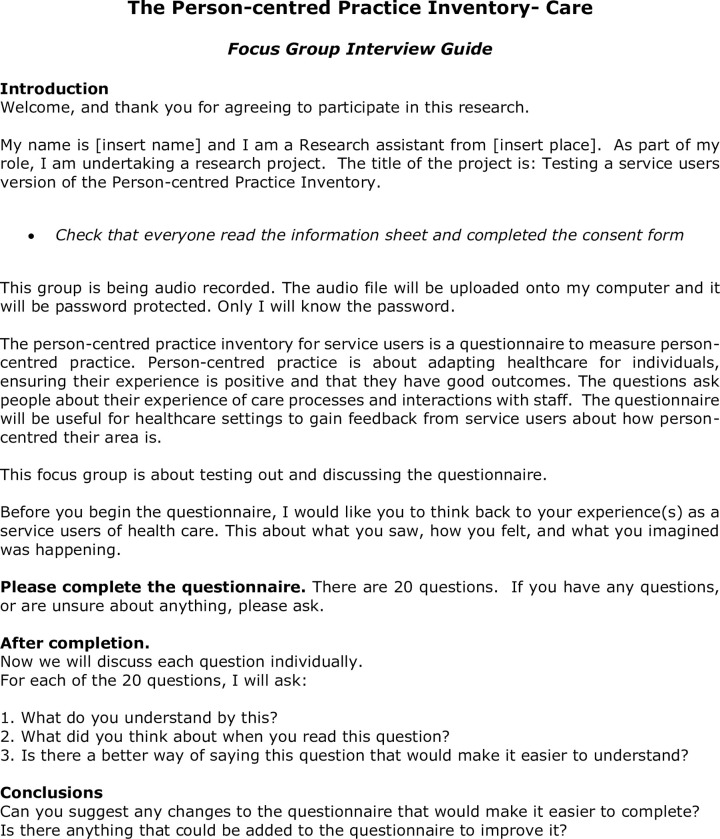
Focus group guide.

A Focus Group guide was developed (Fig 3). Participants were invited to draw on their experiences as a user of the service; particularly remembering what they saw, the way they felt and what they imagined was happening [32]. The participants were invited to independently complete the PCPI-C and the purpose of the exercise was reiterated i.e. to think about how each question of the PCPI-C reflected their own experiences and their answers to the questions. Following completion of the questionnaire, participants were asked to comment on each question in the PCPI-C (20 questions), with a specific focus on their understanding of the question, what they thought about when they read the question, and any suggestions to improve readability. The focus group was concluded with a discussion on the overall usability of the PCPI-C. Each focus group was audiotaped and the audio recordings were transcribed in full. The facilitators of the focus group then listened to the audio recordings, alongside the transcripts, and identified the common issues that arose from the discussions and noted against each of the questions in the draft PCPI-C. Revisions were made to the questions in accordance with the comments and recommendations of the participants. At the end of the analysis phase of each focus group, a table of comments and recommendations mapped to the questions in the instrument was compiled and sent to the whole research team for review and consideration. The comments and recommendations were reviewed by the research team and amendments made to the draft PCPI-C. The amended draft was then used in the next focus group until a final version was agreed. Focus group 1 was held in Scotland, focus group 2 in Australia and focus group 3 in Northern Ireland. Table 2 presents a summary of the feedback from the final focus group.

**Table 2:** PCPI-C results—Round 3: Quotes that demonstrate consensus with the questions or suggests changes.

Questions	Comments	Revisions
1.Staff make an effort to get to understand what is important to me.	*“Trying to find out what’s things that are important to the patient … “* **Too wordy–remove the “to get”** Clear and important question Important question *“They don’t do it all the time”* Patient says to patient in the interview *“You’re from the country*, *they don’t talk to you about the city” “They know what you are there for*? *They should know I’m there for a colonscopy” is this a medical question where is this coming from*?*”* “*Where am I supposed to be to answer this*? *e*.*g*. *ED don’t have time*. *Need the questionnaire to be specific as I moved through many departments”* *“They have too much paperwork and if it’s not documented then it’s not done*!*”*	Change to: *try to understand what is important to me”*
2.Staff use my personal experiences to build a relationship with me	*“I” m not sure of what level of personal experiences its referring to”*. *I don’t think there time to do that with my experience*. *Nurses are always busy*. *Its’ not just nurses is it*? *Drs definitely don’t have time and nurses are wonderful but I don’t think they have time to stand around and talk”* *Are they talking about where you are in life at this stage*? *Are you in a relationship*? *Stuff from way back*? *This is what you would expect of a friend somebody you’re getting to know* *It’s not an expectation of nursing from my perspective*. *Other things in there that might cover this* “*I am not sure of the value*, *what is it they are asking*?*”* *“What personal experience–I don’t understand the context*, *too vague”*. *V2* *I don’t want a life story- personal experience of health not life V1* *Suggested “in relation to your last hospital visit have you had a positive experience or done better*?*”V1* *“You need to be there for a long time to have a relationship”* *“Don’t get to know surgical patients”* *“There is a big turnover of staff”* *“personal experiences–the patient needs to divulge this information”* *“Goes both ways”* *It’s not a “relationship”*. *Group said “rapport” was a better word but then thought maybe not everybody understood this*. *“I don’t feel staff want to build a relationship as such*. *A better question would be How did the staff treat you*? *How did you feel*?*”* *“When there are agency staff in*.* *.* *. *they don’t want to form a relationship with the patient and they’re getting more money than the regular nurses”* *NOTE*: Participants struggled with this question especially those who were admitted for a short time	Keep original
3. Staff involve me in making decisions about my care.	I have written “*Some decisions” as some decisions staff would make without or patients unable to make those decisions*. “You would feel good if involved partly. Not all decisions” *Interviewer*: *Can you give me an example of when staff should take the lead*? *Sometime they have to be firm–get out of bed now and if the patient not feeling like getting out of bed they need to be up and mobile*. *Medications–the nurse may say you need to do this* Change to “some decisions”….they are the experts they know what they are doing. “Staff are there to help me get through this. Deal with the situation” “The general attitude of staff—all are different “ *“I think it’s wrong to be pushed out the next day*. *I got an infection and I had to travel quite a distance every day from home to get it drained*.* *.* *. *you’re not involved in those decisions”*	No change
4. Staff consider my home environment in meeting my care needs	*This is like when you go home for afterwards*? *That’s good*. *You wouldn’t have to do a lot in the home environment but try to find out about home environment …*.*steps*. No change to this question “*They have asked me numerous times who will be at home*, *how will you get there*?*’* *“I have been told no vacuuming” group laughs with patient* “*Best question*” *“Discharge status*, *admission “* *“I had the best consultant in the world*. *He was really person-centred*. *He came every day and spoke to me and asked me who would be at home with me and how would I get home and if I was happy with everything*.*”* Very important	No change
5. Staff give me their full attention when they are with me	An important one *“Like to know they are listening to you”* *“They can’t do anymore and are positive and compassionate* V1 *“Yes when they’re giving you your treatment*, *they check everything and are with you to make sure everything is safe and they watch out for any reactions*.*”* No change	No change
6. I feel able to say to staff what is important to me.	This is minor Change “say to” for “tell the” Important question *“This is very dependent on the nurse your with–their actions”* *“I had a nasty attack of pain and I lay for some time–the patient next to me needed them”* *“but you should be able to say I need you”* *“Is this about medical treatment or personal*? *“* *“Need to add a text box so you can explain answers”* *I have a headache–can you help me”*. *“They give you their full attention and are listening and responding”* V1 **“***There was one particular nurse who shone above all the rest and I literally could tell her anything…she really listened …you always felt she absolutely understood you”*. Request for text boxes to explain answers	No change
7. I feel able to give staff feedback about my experience of being cared for.	“*That’s great and important you can do that with some people but not all people”*. Change to “*I feel comfortable giving*….” “*There might be things happy about and not happy about need to be able to do both of those …*.*people may be more comfortable written…not verbal feedback” (*Advised this was a questionnaire and was written feedback) “You’re usually given a form to feedback but it’s not individualised”. “One night one of the nurses had a bad night-the way she talked to others” “*If you work with one nurse like that how do you answer the question overall”* Suggestion “I feel comfortable” giving staff feedback “ “*When*, *how*, *where…depends on the personalities of staff*” *“If staff ask people who are vulnerable for feedback…like when a patient is receiving chemotherapy …no matter how much the patient may trust them*, *there is a fear that you’re going to say something to someone and it will cause a backlash…”* Important question	No change
8. Staff ask me about my life.	*“It’s nice if they have got time to show interest in the patient*, *but if they haven’t you understand*, *you’re in there because you need to get better…*. *I don’t think they have time to find out a lot about individual patient but in the process of doing work if they ask questions they are showing an interest”* No change in question ***“****not a worthy question again it’s the thing a friend would do*. *I would be happy but wouldn’t expect it”*. *“Dealing with loads of staff–so how do you make a statement–some people do”* *“They don’t have time to be personal “* *“Mainly about my injury”* *“Relates to the home environment–Social workers role*?*”* *“If they ask about the home its “nosey””* *“I don’t like the question it has no valued in it”* *“I don’t mind it–not the most important question–friendliness is important–Privacy is also important you should volunteer information only -don’t want to waste their time*. *It has to be the right timing as I don’t want to distract them* “ *“The majority of them don’t…they don’t have time”* *“They do ask you in the beginning at the first visit… not really after that”*. Can this question be combined with question 9?	No Change/ keep original *“Staff ask me about my life”*
9. Staff connect with me as a person.	*“What does this mean*? *I don’t like wording-what does connect mean*. *Staff listen or take notice to me and that is important*. *You like to feel they are listening and taking notice of your needs”* *“This is like the relationship question*.* *.* *. *I suppose when they have time*, *which isn’t very often*, *they do try to get to know more about you and what’s important to you as a person*.*”*	No change
10. Staff ask me if I have all the information I need	No change–self-explanatory and important *“Very important question especially for family”* *“What if you are sick and can’t be bothered as you are so ill”* *After my operation*, *my diet had to change considerably but the dietician didn’t come to see me for 4 days…I had porridge on the first day and was violently ill…they thought it was the anaesthetic*! *When the dietician came 4 days later*, *she said I should never have had porridge*!	No change
11 Staff don’t assume they know what is best for me.	“*They don’t come and tell you …*.*they take notice and involve you in the decisions* *I wrote down “Staff don’t tell me what’s best for me”* *“Nice to feel your listened to”* Yes the nurse ask how are my pain levels they don’t assume What use is this question? I don’t have a clear wayV1 Not a negative “don’t” in a question V2 Staff know what’s best for me if medical.V1 They help me if I have questions–they give information I need V2 *“The question is straightforward enough and easy enough for people to answer”*	No change
12. When we don’t agree about my care staff try to find common ground	*This assumes this will happen change to “if we don’t…*.*” need a not applicable box* *“When” says there will always be time…lots of times not going to happen* *Great if that’s what can happen* *Comma required between care*, *staff try to find common ground or compromise (either or)* *Nice to think you can have care and no conflict*. *Who is “we”*? *We don’t agree- could say “disagree”*. *The words are confusing*. *This applies to “wingey” patients only (*? *Australian word- other word would be complainers)* *“The Patient across the bed*, *nurses forgot to put patient only on their back*, *the patient told the other nurse who apologised”* *“Bedside handover good time for agreeing about care”* *Perhaps “when we disagree about care…* *May mean when you get out of bed and the team have to coerce you*. *Like food and exercise* *“if” only I had that experience where we have not agreed- I would leave it neutral*? *“This isn’t going to happen* *- Nurses and drs have opinions*. *I want to keep my eye and I don’t have the knowledge to make decisions*. *I need to trust*. *I won’t disagree”* *“When you’re admitted to the chemotherapy unit*, *you feel far too vulnerable to get into a discussion about care*.* *.* *.*you’re just so grateful that something can be done to help you”*	Change to: *“When we disagree about my care*, *staff try to find common ground”*
13. My family are included in decisions about my care when I want them to be.	Important especially for going home *“The family should always be involved*” Straightforward question Change to … “*My family are included in decisions about my care only when I want them to be*”. (highlight want them to be) *Exclusion to this would be dementia*. “*I want to be the one who makes decisions”* *“It’s important to have a family member in with you*, *particularly at the beginning…* *when you’re diagnosed and they’re explaining what’s going to happen… treatment and that*.* *.* *.*you get it hard to remember all that they told you and you definitely need someone there”*	Change to: *“My family are included in decisions about my care only when I want them to be”*
14. Staff use what they know about me as a person in my care	Not well worded…. *“In looking after someone they find out something and use that”* I didn’t get an alternate to this ….possibly “*In caring for me*, *staff use what they know about me as a person”…*.easier to understand *In the start they won’t know it depends on how long you are there for* Good but what if they don’t know anything about me	Change to: *“In caring for me*, *staff use what they know about me as a person”*
15. I feel cared for	Suggestion—*I feel the staff care about me* *It seems to say a bit better what it’s trying to say* *You might not want to hear all these comments* *Important question–”can you explain further if strongly disagreeing*. *They could be amazing” V2* *Couple of questions need that extra section to write comments for the moral of staff V1* *“I was very unwell after my chemo and in and out of hospital*. *At one stage*, *I was so ill*, *I literally crawled to the nurse’s station for a pain killer… the nurse said you have 15 more minutes before you’re due it*. *A son-in-law of another patient lifted me back to bed and that nurse went off duty and I still didn’t get the pain killer*. *When the night nurse came on duty she had to get the doctor because the nurse who went off had filled in that I had got my pain killer*. *The man who lifted me back to bed was able to say exactly what happened”*.	No change
16. Staff respond compassionately when I am upset or unhappy	*“It nice to think you can have a good time in hospital and not be upset”* Change to “*staff respond compassionately if I am upset or unhappy”* *Also need a Not Applicable box* for all of them *“Nobody wants to be here”* *“Glad to get out “* *“It’s not just about nursing care*, *its personal and emotional etc*. *People are emotional”*	No change
17. Staff help me to express my concerns about my treatment and care.	No change *“I hope they do*, *according to where you are*. *Drs do better than nurses except for a couple*.. *young doctors better*” V2	No change
18. Staff listen to me and hear what I have to say about my care	Important–doesn’t need the two bits. Could remove “me and hear”. Remove hear and write understand *“It’s a good question*. *I hear you sometimes goes in one ear and out the other” V2* “Active listen is the word isn’t it?” *“This doesn’t always happen”*.	No Change
19. Staff understand my family circumstances when caring for me.	*“Great if they have got time for doing that”*. *“By the time you go home they fully understand”*. *“Social economic factors”* *“Some get more from their families”* *“House safe–I can’t use the shower”* *“I’ m going home and my parents will come around”* *“Hospital in the Home are great”* *Some get more from families than others* *SW needs to help* Good questions– *“How do you get to know about me*?” *“I feel they usually always get to know and understand before you’re discharged home”*	No change
20. Staff help me to set realistic goals	“*Meaningful of getting better*, *going home…*.*is that what it’s referring to*?*”* *Realistic is an important word*. Interviewer discussed how goals could be set at any time for anything she *replied “I wasn’t sure…*..*until you explained to me”* Suggestions: Maybe staff help me set realistic daily goals or personal or physical goals for me” *Needs more explanation as I wasn’t sure*.	No change

A final stage of development involved distributing the agreed version of the PCPI-C to members of ‘The International Community of Practice for Person-centred Practice’ (PcP-ICoP) for review and feedback. The PcP-ICoP is an international community of higher education, health and care organisations and individuals who are committed to advancing knowledge in the field of person-centredness. No significant changes to the distributed version were suggested by the PcP-ICoP members, but several members requested permission to translate the instrument into their national language. PcP-ICoP members at the University of Vienna, who were leading on a large research project with nursing homes in the region of Lower Austria, agreed to undertake a parallel translation project as a priority, so they could use the PCPI-C in their research project. The instrument was culturally and linguistically adapted to the nursing home setting in an iterative process by the Austrian research team in collaboration with the international research team. Data were collected through face-to-face interviews by trained research staff. Residents of five nursing homes for older persons in Lower Austria were included. All residents who did not have a cognitive impairment or were physically unable to complete the questionnaire (because of ill-health) (n = 235) were included. 71% of these residents (N = 167) managed to complete the questionnaire. Whilst in Austria, formal ethical approval for non-intervention studies is not required, the team sought informed consent from participants. Particular attention was paid throughout the interviews to assure ongoing consent of residents by carefully guided conversations.

### Phase 3: Testing structural validity of the PCPI-C

The aim of this phase was to test the structural validity of the PCPI-C using confirmatory factor analysis with an international sample of service users. The PCPI-C comprises 20 items measured on a 5-point scale ranging from ‘strongly disagree’ to ‘strongly agree. The 20 items represent the 5 constructs comprising the final model to be tested, which is outlined in Table 3.

**Table 3:** Descriptive statistics and factor loadings for the PCPI-C.

Statement mapped to Person-centred Processes	Item No	Mean	SD	skewness	Kurtosis	Loading
**Working with the Person’s Belief and Values (alpha = .79)**						
Staff make an effort to understand what is important to me	1	3.46	0.79	-1.65	2.88	0.74*
In caring for me, staff use what they know about me as a person	14	3.01	1.01	-0.86	.12	0.65*
I feel able to give staff feedback about my experience of being cared for	7	3.33	0.87	-1.52	2.51	0.71*
I feel able to say to staff what is important to me	6	3.51	0.74	-1.89	4.40	0.64*
**Sharing Decision-making (alpha = 0.70)**						
Staff involve me in making decisions about my care	3	3.13	1.0	-1.18	0.94	0.72*
Staff help me to express my concerns about my treatment and care	17	3.24	0.90	-1.29	1.45	0.73*
My family are included in decisions about my care only when I want them to be	13	2.80	1.11	-.069	-0.15	0.39*
Staff help me to set realistic goals	20	3.10	0.96	-0.94	0.41	0.77*
Staff ask me if I have all the information, I need	10	3.15	1.03	-1.48	1.96	0.72*
**Engaging Authentically (alpha = 0.74)**						
When we disagree about my care, staff try to find common ground	12	2.98	0.94	-0.51	-0.24	0.60*
Staff listen to me and hear what I have to say about my care	18	3.34	0.85	-1.57	2.90	0.74*
Staff don’t assume they know what is best for me	11	2.42	1.29	-.035	-0.96	0.30*
Staff connect with me as a person	9	3.17	0.96	-0.47	-.077	0.69*
**Being Sympathetically Present (alpha = 0.66)**						
Staff respond compassionately when I am upset or unhappy	16	3.18	0.94	-1.08	0.76	0.61*
Staff give me their full attention when they are with me	5	3.63	0.73	-2.50	6.94	0.69*
Staff use my personal experiences to build a relationship with me	2	3.05	0.99	-1.00	0.57	0.69*
**Working Holistically (alpha = 0.79)**						
I feel cared for	15	3.55	0.76	-2.24	6.07	0.74*
Staff ask me about my life	8	2.50	1.26	-0.47	-0.77	0.55*
Staff consider my home environment in meeting my care needs	4	2.99	1.0	-0.64	-0.25	0.67*
Staff understand my family circumstances when caring for me	19	3.04	0.98	-0.82	0.24	0.69*

* Denotes statistically significant p>0.01.

### Sampling

A sample of 452 participants was selected for this phase of the study. The sample selected comprised two groups. Group 1 (n = 285) were service users with cancer (breast, urological and other) receiving radiotherapy in four Cancer Treatment Centres in four European Countries–UK, Malta, Poland and Portugal. These service users were participants in a wider SAFE EUROPE (www.safeeurope.eu) project exploring the education and professional migration of therapeutic radiographers in the European Union. In the UK a study information poster with a link to the PCPI-C via Qualtrics^©^ survey was disseminated via UK cancer charity social media websites. Service user information and consent were embedded in the online survey and presented to the participant following the study link. At the non-UK sites, hard copy English versions of the surveys were available in clinical departments where a convenience sampling approach was used, inviting everyone in their final few days of radiotherapy to participate. The ‘DeepL Translator’ software (DeepL GmbH, Cologne, Germany) was used to make the necessary terminology adaptions for both the questionnaire and the participant information sheet across the various countries. Fluent speakers based in the participating sites and who were members of the SAFE EUROPE project team confirmed the accuracy of this process by checking the accuracy of the translated version against the original English version. Participants were provided with study information and had at least 24 hours to decide if they wished to participate. Willing participants were then invited to provide written informed consent by the local study researcher. The study researcher provided the hard copy survey to the service user but did not engage with or assist them during completion. Service users were informed they could take the survey home for completion if they wished. Completed surveys were returned to a drop box in the department or returned by post (data collected May 2021-March 2022). Group 2 were residents in nursing homes in Lower Austria (n = 125). No participating residents had a cognitive impairment and were physically able to complete the questionnaire. Data were collected through face-to-face interviews by trained research staff (data collected January 2021-March 2021).

### Statistical analysis

Descriptive and measures of dispersion statistics were generated for all items to help inform subsequent analysis. Measures of appropriateness to conduct factor analysis were conducted using The Kaiser-Meyer-Olkin Measures of Sampling Adequacy and Bartletts Test of Sphericity. Inter-item correlations were generated to examine for collinearity prior to full analysis. Confirmatory factor analysis was conducted using maximum likelihood robust extraction testing of the 5-factor model.

Acceptable fit statistics were set at Root Mean Square Estimations of Approximation (RMSEA) of 0.05 or below; 90% RMSEA higher bracket below 0.08; and Confirmation Fit Indices (CFI) of 0.95 or higher and SRMR below 0.05 [33–35]. Internal consistency was measured using Cronbach alpha scores for factors in the accepted factor model.

The model was re-specified using the modification indices provided in the statistical output until acceptable and a statistically significant relationship was identified. All re-specifications of the model were guided by principles of (1) meaningfulness (a clear theoretical rationale); (2) transitivity (if A is correlated to B, and B correlated to C, then A should correlate with C); and (3) generality (if there is a reason for correlating the errors between one pair of errors, then all pairs for which that reason applies should also be correlated) [36].

Acceptance modification criteria of:

1. The items to first order factors were initially fitted.
2. Correlated error variance permitted as all items were measuring the same unidimensional construct.
3. Only statistically significant relationship retained to help produce as parsimonious a model as possible.
4. Factor loadings above 0.40 to provide a strong emergent factor structure.

Factor loading scores were based on Comrey and Lee’s [37] guidelines (>.71 = excellent, >.63 = very good, >.55 = good, >.45 = fair and >.32 = poor) and acceptable factor loading given the sample size (n = 452) were set at >0.3 [33,38].

## Results and discussion

### Demographic details

The sample of 452 participants represented an international sample of respondents drawn from across five countries: UK (14.6% n = 66), Portugal (47.8%. n = 216), Austria (27.7%, n = 125), Malta (6.6, n = 30) and Poland (3.3%, n = 15). Table 4 outline the demographic characteristics of the sample. The final sample of 452 participants provides an acceptable ratio^33^ of 22:1 respondent to items.

**Table 4:** Demographic characteristic of the sample.

Country	Gender			Age		Conditions	
	Female	Male		18–24	1.2%	Nursing Home	27.7%
**UK (14.6%, n = 66)**	**13.7% n = 62**	**0.9 n = 4**		**25–34**	**7.9%**	**Breast cancer**	**20.4%**
**Portugal (47.8%, n = 216)**	**26.3% n = 119**	**21.5% n = 97**		**45–54**	**17.9%**	**Head and Neck Cancer**	**3.3%**
**Austria (27.7%, n = 125)**	**21.7% n = 152**	**6.0%, n = 27**		**55–64**	**17.9%**	**Prostate Cancer**	**13.4%**
**Malta (6.6%, n = 30)**	**2.9% n = 13**	**3.8% n = 17**		**65+**	**55%**	**Other Cancers**	**35.2%**
**Poland (3.3%, n = 15)**	**1.8% n = 8**	**1.5% n = 7**					
**All n = 452**	**66.4% n = 300**	**33.6% n = 152**					

The means scores indicate that respondents scored the items neutrally. The measures of skewness and kurtosis were acceptable and satisfied the conditions of normality of distribution for further psychometric testing. Examination of the Kaiser Meyer Olkin (0.947) and the Bartlett test for sphericity (4431.68, df = 190, p = 0.00) indicated acceptability of performing factor analysis on the items. Cronbach alpha scores for each of the constructs confirm the acceptability and unidimensionality of each construct.

**Fig 4:**
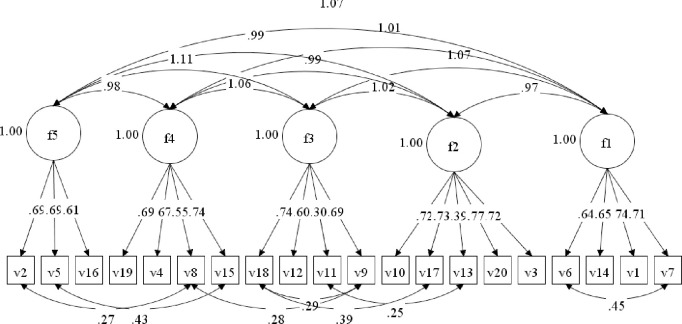
Structural model of the PCPI-C.

Examination of the correlation matrix between items shows a range of between 0.144 and 0.740, indicating a broadness in the areas of care the questionnaire items address, as well as no issues of collinearity. The original measurement model was examined using maximum likelihood extraction and the original model had mixed fit statistics. All factor loadings (except for items 11 and 13) were above the threshold of 0.4 (Table 3). Six further modifications were introduced into the original model based on highest scored modification indices until the fit statistics were deemed acceptable (Table 5 for model fit statistics and Fig 4 for items correlated errors). Two item correlated error modifications were within factors and 4 between factors. The accepted model factor structure is displayed in Fig 4.

**Table 5:** Fit statistics for the PCPI-C models (before and after model respecification).

	Chi Square	RMSEA	90% RMSEA	CFI	SRMR
**Original Model**	529/160	0.072	0.065–0.079	0.87	0.058
**Fitted Model**	341/153	0.053	0.045–0.060	0.93	0.048

The results demonstrate that the PCPI-C is a psychometrically sound instrument, supporting a five-factor model that examines service user perspectives of what constitutes person-centred care.

## Discussion

Measuring person-centred care is a complex and challenging endeavour [39]. In a review of existing measures of person-centred care, DeSilva [39] identified that whilst there are many tools available to measure person-centred care, there was no agreement about which tools were most worthwhile. The complexity of measurement is further reinforced by the multiplicity of terms used that imply a person-centred approach being adopted without explicitly setting out the meaning of the term. Further, person-centred care is multifaceted and comprises a multitude of methods that are held together by a common philosophy of care and organisational goals that focus on service users having the best possible (personalised) experience of care. As DeSilva suggested, *“it is a priority to understand what ‘person-centred’ means*. *Until we know what we want to achieve*, *it is difficult to know the most appropriate way to measure it*. *(p 3)”*. However, it remains the case that many of the methods adopted are poorly specified and not embedded in clear conceptual or theoretical frameworks [40,41]. A clear advantage of the study reported here is that the PCPI-C is embedded in a theoretical framework of person-centredness (the PCPF) that clearly defines what we mean by person-centred practice. The PCPI-C is explicitly informed by the ‘person-centred processes’ domain of the PCPF, which has an explicit focus on the care processes used by healthcare workers in providing healthcare to service-users.

In the development of the PCPI-C, initial items were selected from the Person-centred Practice Inventory-Staff (PCPI-S) and these items are directly connected with the person-centred processes domain of the PCPF. The PCPI-S has been translated, validated and adopted internationally [23–28] and so provides a robust theoretically informed starting point for the development of the PCPI-C. This starting point contributed to the initial acceptability of the instrument to participants in the focus groups. Like DeSilva, [39] McCormack et al [42] and McCormack [41] have argued that measuring person-centred care as an isolated activity from the evaluation of the impact of contextual factors on the care experienced, is a limited exercise. As McCormack [41] suggests “*Evaluating person-centred care as a specific intervention or group of interventions*, *without understanding the impact of these cultural and contextual factors*, *does little to inform the quality of a service*.*” (p1)* Using the PCPI-C alongside other instruments such as the PCPI-S helps to generate contrasting perspectives from healthcare providers and healthcare service users, informed by clear definitions of terms that can be integrated in quality improvement and practice development programmes. The development of the PCPI-C was conducted in line with good practice guidelines in instrument development [29] and underpinned by an internationally recognised person-centred practice theoretical framework, the PCPF [5]. The PCPI-C provides a psychometrically robust tool to measure service users’ perspectives of person-centred care as an integrated and multi-faceted approach to evaluating person-centredness more generally in healthcare organisations.

With the advancement of Patient Reported Outcome Measures (PROMS) [43,44], Patient Reported Experience Measures (PREMS) [45] and the World Health Organization (WHO) [15] emphasis on the development of people-centred and integrated health systems, greater emphasis has been placed on developing measures to determine the person-centredness of care experienced by service users. Several instruments have been developed to measure the effectiveness of person-centred care in specific services, such as mental health [45], primary care [46,47], aged care [48,49] and community care [50]. However only one other instrument adopts a generic approach to evaluating services users’ experiences of person-centred care [51]. The work of Fridberg et al (The Generic Person-centred Care Questionnaire (GPCCQ)) is located in the Gothenburg Centre for Person-centred Care (GPCC) concept of person-centredness—patient narrative, partnership and documentation. Whilst there are clear connections between the GPCCQ and the PCPI-C, a strength of the PCPI-C is that it is set in a broader system of evaluation that views person-centredness as a whole system issue, with all parts of the system needing to be consistent in concepts used, definitions of terms and approaches to evaluation. Whilst the PCPI-S evaluates how person-centredness is perceived at different levels of the organisation, using the same theoretical framework and the same definition of terms, the PCPI-C brings a service user perspective to an organisation-wide evaluation framework.

A clear strength of this study lies in the methods engaged in phase 2. Capturing service user experiences of healthcare has become an important part of the evaluation of effectiveness. Service user experience evaluation methodologies adopt a variety of methods that aim to capture key transferrable themes across patient populations, supported by granular detail of individual specific experience [43]. This kind of service evaluation depends on systematically capturing a variety of experiences across different service-user groups. In the research reported here, service users from a variety of services including palliative care and cancer services from three countries, engaged in the focus group discussions and were freely able to discuss their experiences of care and consider them in the context of the questionnaire items. The use of focus groups in three different countries enabled different cultural perspectives to be considered in the way participants engaged with discussions and considered the relevance of items and their wording. The sequential approach enabled three rounds of refinement of the items and this enabled the most relevant wording to be achieved. The range of comments and depth of feedback prevented ‘knee-jerk’ changes being made based on one-off comments, but instead, it was possible to compare and contrast the comments and feedback and achieve a more considered outcome. The cultural relevance of the instrument was reinforced through the translation of the instrument to the German language in Austria, as few changes were made to the original wording in the translation process. This approach combined the capturing of individual lived experience with the systematic generation of key themes that can assist with the systematic evaluation of healthcare services. Further, adopting this approach provides a degree of confidence to users of the PCPI-C that it represents real service-user experiences.

The factorial validity of the instrument was supported by the findings of the study. The modified models fit indices suggest a good model fit for the sample [31,34,35]. The Confirmation Fit Indices (CFI) fall short of the threshold of >0.95. However, this is above 0.93 which is considered an acceptable level of fit [52]. Examination of the alpha scores confirm the reliability (internal consistency) of each construct [53]. All factor loadings were at a statistically significant level and above the acceptable criteria of 0.3 recommended for the sample size [38]. All but 2 of the loadings (v11 –‘*Staff don’t assume they know what is best for me’* and v13 –*‘My family are included in decisions about my care only when I want them to be’*) were above the loadings considered as good to excellent [37]. At the level of construct, previous research by McCance et al [54] showed that all five constructs of the person-centred processes domain of the Person-centred Practice Framework carried equal significance in shaping how person-centred practice is delivered, and this is borne out by the approval of a 5-factor model in this study. However, it is also probable that there is a degree of overlap between items across the constructs, reflected in the 2 items with lower loadings. Other items in the PCPI-C address perspectives on shared decision-making and family engagement and thus it was concluded that based on the theoretical model and statistical analysis, these 2 items could be removed without compromising the comprehensiveness of the scale, resulting in a final 18-item version of the PCPI-C (available on request).

Whilst a systematic approach to the development of the PCPI-C was adopted, and we engaged with service users in several care settings in different countries, further research is required in the psychometric testing of the instrument across differing conditions, settings and with culturally diverse samples. Whilst the sample does provide an acceptable respondent to item ratio, and the sample contains international respondents, the model structure is not examined across international settings. Likewise, further research is required across service users with differing conditions and clinical settings. Whilst this is a limitation of this study reported here, the psychometric testing of an instrument is a continuous process and further testing of the PCPI-C is welcomed.

## Conclusions

This paper has presented the systematic approach adopted to develop and test a theoretically informed instrument for measuring service users’ perspectives of person-centred care. The instrument is one of the first that is generic and theory-informed, enabling it to be applied as part of a comprehensive and integrated framework of evaluation at different levels of healthcare organisations. Whilst the instrument has good statistical properties, ongoing testing is recommended.
