# Correction: The development and structural validity testing of the Person-centred Practice Inventory–Care (PCPI-C)

**DOI:** 10.1371/journal.pone.0322949

**Published:** 2025-04-22

**Authors:** Brendan George McCormack, Paul F. Slater, Fiona Gilmour, Denise Edgar, Stefan Gschwenter, Sonyia McFadden, Ciara Hughes, Val Wilson, Tanya McCance

In the Phase 1 – Item selection subsection of the Materials and methods, there is an error in the first sentence. The correct sentence is: An initial item pool for the PCPI-C was identified by BGMcC and TMcC by selecting items from the ‘person-centred processes’ sub-scale of the PCPI-S (Table 1).
